# Syntheses of a Hyperbranched Polymer and Its Performance on Enhanced Oil Recovery

**DOI:** 10.3389/fchem.2021.738717

**Published:** 2021-11-05

**Authors:** Sanyuan Qiao, Qingwang Liu, Xian Zhang, Hongchang Che

**Affiliations:** ^1^ Petroleum Engineering, Northeast Petroleum University, Daqing, China; ^2^ Wells and Operation Department, PetroChina International Middle East, Beijing, China

**Keywords:** syntheses, hyperbranched polymer, surfactant, core flooding, enhanced oil recovery

## Abstract

A hyperbranched carboxylate-type polymer was synthesized through esterification and carboxymethylation, and its performance on enhanced oil recovery was experimentally evaluated. The optimum condition for esterification was 8 h at 120°C, where 3% PTSA as the catalyst and 9:1 mol ratio of the AB_2_ intermediate and trimethylolpropane were used. The optimum condition for carboxymethylation was 4 h at 80°C. The critical micelle concentration of the hyperbranched polymer was 433.63 mg/L, the Krafft point was 5°C, and the surface tension was lowered to 28 mN/m. In the range of 400–500 mg/L concentration, the adsorption onto the oil sand surface achieved equilibrium, and micellar solubilization reached 600 ml/mol. The interfacial tension can be lowered to a level of 10^−2 ^mN/m by the single use of the hyperbranched polymer, and the value further decreased to a level of 10^−3 ^mN/m while being formulated with sodium dodecylsulfate or NaOH. Oil recovery of water flooding was further enhanced by the single use of a hyperbranched polymer or the combination of hyperbranched polymer/sodium dodecylsulfate. The latter exhibited more prosperous advantages in low-permeability reservoirs.

## Introduction

Polymer flooding is an economic and efficient method for enhanced oil recovery and is used worldwide (Zhao et al., 2020; Sieberer et al., 2017). Saboorian-Jooybari et al. presented a detailed review of the application of polymers in heavy oil flooding since the 1960s and concluded that polymer flooding can contribute to a range of 2.2–44% incremental oil recovery (Saboorian-Jooybari et al., 2016). Sheng et al. discussed the status of polymer flooding technology and highlighted some associated problems such as formation damage and low injectivity of polymers (Sheng et al., 2015). Brattekås and Seright investigated improved polymer gel conformance control during water flooding in fractured low-permeability carbonates and found that gel-blocking efficiency was dependent on water salinity, core materials, and oil presence (Brattekås and Seright, 2018; Wang and Zhang, 2019). Algharaib et al. experimentally investigated the impact of various parameters on polymer performance on enhanced oil recovery and found pre-flush and polymer characteristics having various degrees of influence (Algharaib et al., 2014). Cardoso et al. discussed the importance of chemical characteristics of polymers in enhanced oil recovery processes and concluded that more profitable results can be gained using polymers of higher average molar mass in the semidilute regime (Cardoso et al., 2016).

Hyperbranched polymers (HBPs) are highly branched macromolecules with a three-dimensional dendritic architecture, and a wide range of applications have been reported due to their well-defined structures, unique properties, and facile synthesis (Gurunathan et al., 2016). They usually have a large degree of functional terminal groups and intramolecular cavities presenting high solubility and low viscosities (Frey, 2003; Caminade et al., 2015).

Flory discussed the possibility of HBP syntheses by polycondensation of the AB_x_-type monomer, to which attention was not paid at the time (Flory, 1941; Flory, 1952). The studies on the topic have thrived since 1990s, and their applications have been extended to enhanced oil recovery (Algharaib et al., 2014; Sun et al., 2016; Yin and Zhao, 2017). Addition of HBPs leads to greater oil recovery than conventional polymers in core flooding experiments (Luo et al., 2019; Jianbo et al., 2020; Chen et al., 2020).

Carboxylate surfactants, derived from petroleum, have been widely used in tertiary oil recovery due to their low adsorption onto sands, strong emulsifying property, and ability of lowering interfacial tension (Ziyuan et al., 2021). HBPs have high interfacial activity and low critical micelle concentration (CMC) and can be modified through abundant terminal groups in the unique structures (Clarke et al., 2016; Khorsandi et al., 2017).

This article is intended to synthesize a hyperbranched carboxylate–type polymer and evaluate its performance on enhanced oil recovery.

## Materials and Methods

### Synthesis of Branched Monomers

**FIGURE 1:**
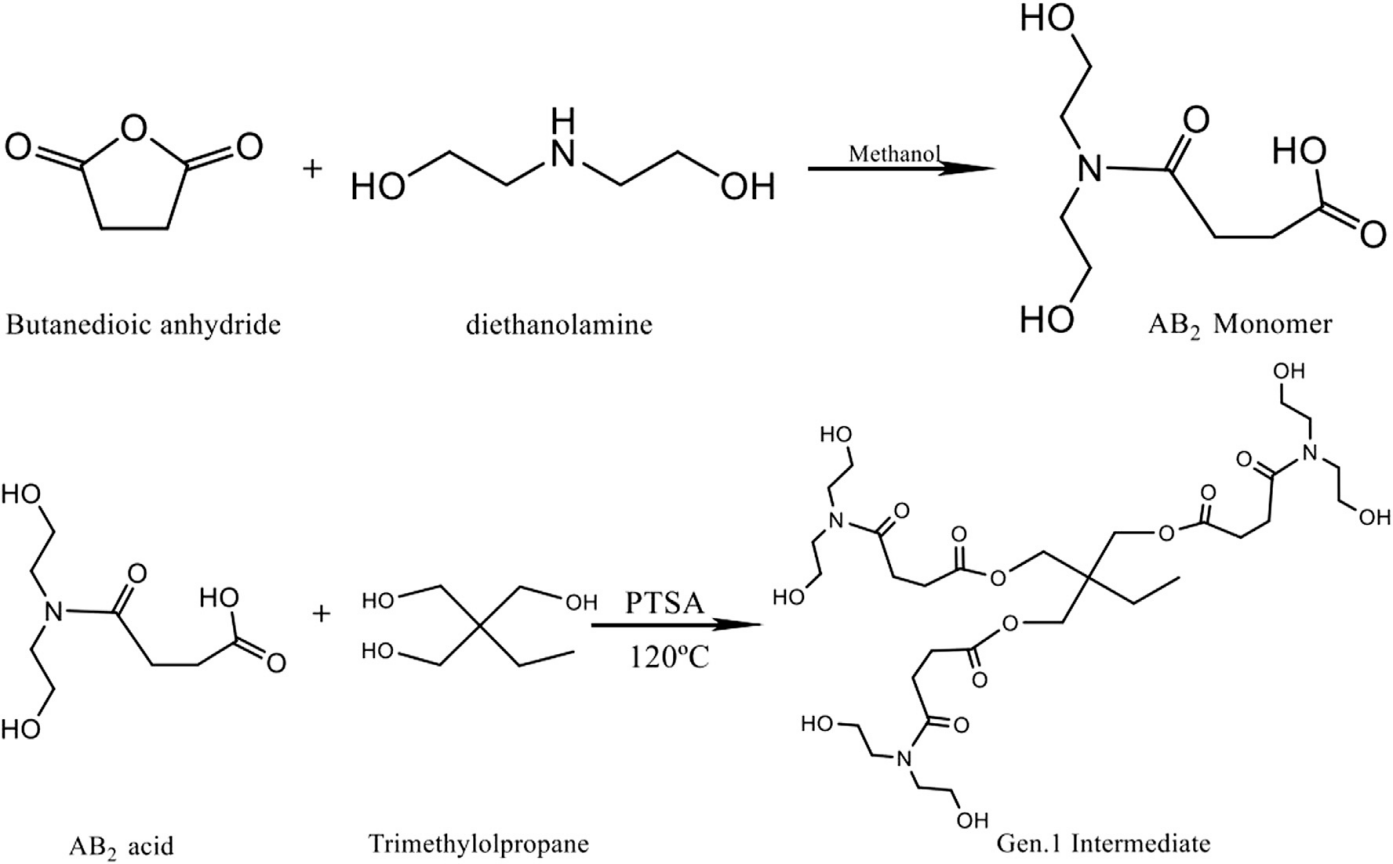
Synthesis of the AB_2_ monomer and Gen. 1 intermediate.

A wide variety of polymer types can be synthesized, and special properties can be imparted via different routes and suitable end capping reactions. In this article, the hyperbranched macromolecules were synthesized by the polymerization of an intermediate (AB_x_ or latent AB_x_ monomers). First, a diethanolamine methanol solution was prepared by dissolving diethanolamine in a methanol solution. Then, succinic anhydride was added into anhydrous methanol and stirred until it was completely dissolved. Then, the diethanolamine solution was added to the succinic anhydride solution, and the final molar ratio of diethanolamine to succinic anhydride was found to be 1:1. The solution was placed in a cold water bath and maintained for 6 h, and then the methanol solution was removed using a rotary evaporator to obtain the AB_2_-type monomer intermediate. Its molecular structure is shown in Figure 1.

### Synthesis of a Gen.1 Intermediate

TMP (trimethylolpropane) is esterified with the AB_2_ monomer at 120°C in the presence of *p*-toluenesulfonamide (PTSA) as the catalyst. The three terminal hydroxyl groups of TMP are equally replaced by the AB_2_ monomer in the reactions, and then the Gen. 1 intermediate is obtained. When each hydroxyl group of TMP is substituted by esterification, two hydroxyl groups are introduced into the end position, and the esterification reactions continue to proceed with the monomer. In order to minimize the consumption of the Gen.1 intermediate by the AB_2_ monomer and assure the equal substitution of terminal hydroxyl groups in TMP, a molar ratio of 1:3 (TMP to the intermediate) is used, and the yield is about 75%. The reaction scheme is shown in Figure 1.

### Synthesis of a Gen.2 Hyperbranched Molecule

**FIGURE 2:**
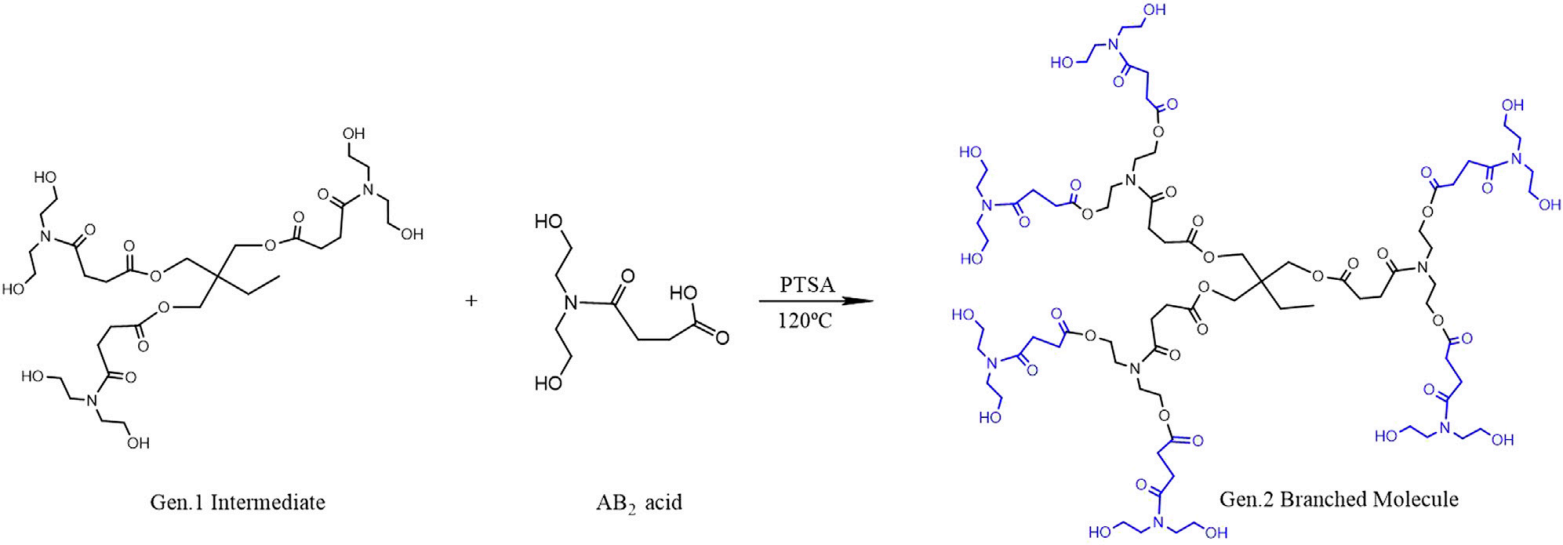
Synthesis of Gen. 2 hyperbranched polyurethane (HPAE).

The Gen.1 intermediate is esterified with the AB_2_ monomer at 120°C in the presence of PTSA as the catalyst. The six terminal hydroxyl groups of the Gen.1 intermediate are esterified with the AB_2_ intermediate, and then the second generation hyperbranched polyurethane (Gen.2 HPAE) is obtained. In the experiments, the molar ratio of 1:6 (Gen.1 intermediate to AB_2_ monomer) is used to minimize the side reactions, and the yield is about 65%. The reaction scheme is shown in Figure 2.

### Synthesis of HBP With Sodium Carboxylate at the End of Gen.2

**FIGURE 3:**
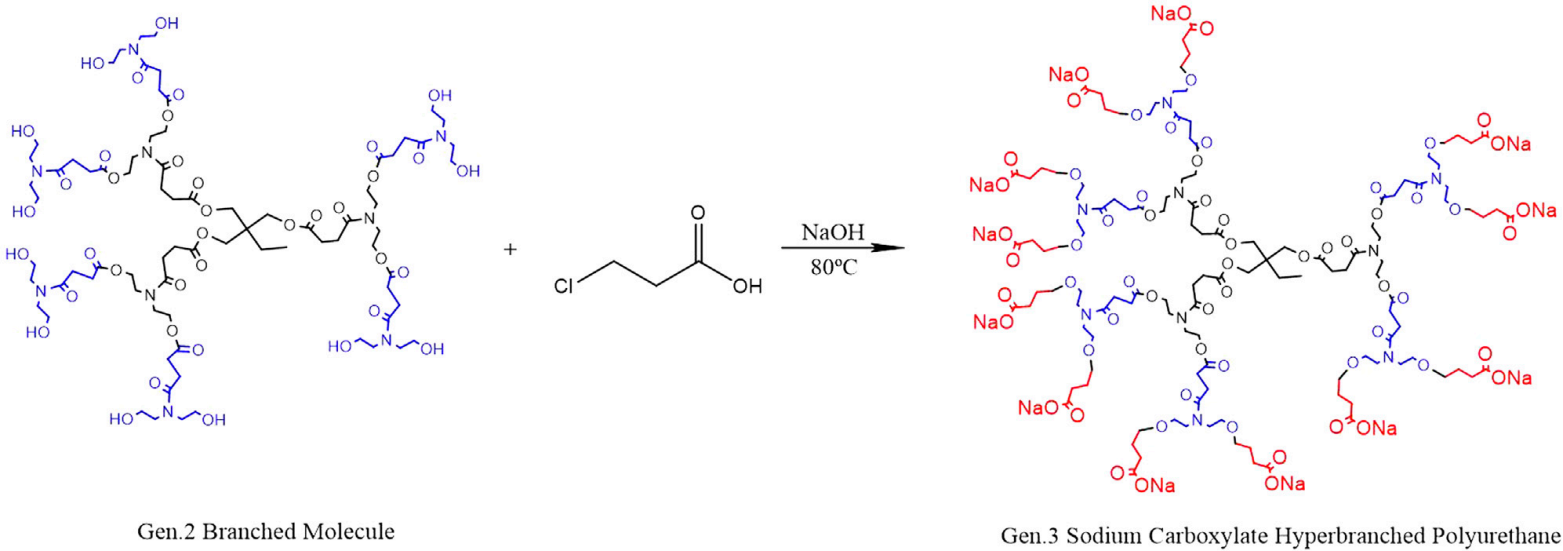
Synthesis of HPAE with sodium carboxylate at the end of Gen.2 (*m*-HPAE).

Modified hyperbranched polyurethane (*m*-HPAE) and NaOH were put into a four-port bottle and heated to 80–100°C under magnetic stirring. A 3-chloropropionic acid solution (80 wt%) was vacuumed for 30–50 min and then poured into the bottle, leaving the reaction to proceed for 4–6 h. The solvent in the system was removed, and the product was washed using anhydrous ethanol and then filtered. The residues with low boiling point were removed using a rotary evaporator and sodium carboxylate–terminated HPAE (SCHP) was obtained. The reaction scheme is shown in Figure 3. In the process of synthesis, the molar ratio (hyperbranched polyurethane to 3-chloropropionic acid and NaOH) was determined to be 1:12:12 to replace 12 terminal hydroxyl groups in the synthesized HPAE.

### Evaluation of the Synthesized HBP

#### Fourier Transform Infrared Spectroscopy

Fourier transform infrared spectroscopy (FT-IR) analyses of the original polymer and the modified HPAE were performed using a VERTE70 (Germany) device. The samples were scanned in the mid-infrared region of wavenumbers (400–4,000 cm^−1^) to record the FT-IR spectra.

### Surface Tension

Surface tension was determined using the ring method where a ring attached to a torsion meter was dipped into the hyperbranched carboxylate surfactant solution. The liquid level was lowered after slow pull-up, and the force on the ring was measured before the liquid film tore off. The surface tension was calculated based on the diameter of the ring and the tear-off force.

### Critical Micelle Concentration and Krafft Point

The CMC was determined by a conductivity method where a conductimeter was used to measure the conductivity of the solution. The Krafft point is the minimum temperature at which surfactants form micelles, that is, micelles cannot be formed below this critical micelle temperature. At the Krafft point, the solubility is equal to the CMC. The Krafft point was measured in the experiment. The solution of *m*-HPAE with a concentration of 1% was prepared and poured into a test tube which was heated in a water bath and stirred until the solution became clear and transparent. Then, it was placed in a cold water bath to allow the temperature to drop under continuous stirring until the precipitation was observed. The measurements were repeated, and the temperature was recorded.

### Adsorption Property

The adsorption property of *m*-HPAE is a significant parameter to determine its suitability for enhanced oil recovery, relevant to its ability and effectiveness to reduce surface tension at the oil–water interface and strip off oil drops from the rock surface. *m*-HPAE varying from 100 mg/L to 900 mg/L was prepared and mixed with 2 g oil sands (from Daqing Oilfield in China) at the liquid/solid ratio of 60:1. The container was placed at 45°C and was continuously shaken for 8 h. The clear solution was obtained after separation and still stratification, and the concentration of the hyperbranched surfactants was determined by phase titration using Bromocresol green based on the following equation.

$$\text{M}=\frac{\left(C_{1}-C_{2}\right)*V}{m},$$

where m is the weight of the oil sands, g; C_1_ is the concentration before adsorption, mg/L; C_2_ is the concentration after adsorption, mg/L; V is the volume of *m*-HPAE, mL; and M is the adsorption capacity, mg/L.

### Micellar Solubilization

Micellar solubilization is the process of taking the solubilizate onto or into micelles as a part of the whole system. In the experiment, the hyperbranched surfactant solution was prepared with a concentration greater than the CMC. 10 ml distilled water was added into seven 50-ml bottles containing benzene with various volumes (10, 20, 30, 40, 50, 60, and 70 μl). The bottles were placed in the water bath at 40°C for 15 min, then taken out, and cooled down to atmospheric temperature. The absorbance value of each solution was measured using a spectrophotometer, and the solubilization value was calculated based on the following equation.

$$\text{Z}=\frac{1000A}{cV},$$

where Z is the solubilizing ability, mL/mol; c is the hyperbranched molecule solution concentration, mol/L; V is the hyperbranched molecule solution volume, mL; and A is the benzene content when reaching the solubilization limit, mL.

### Wettability

Liquid droplets on the solid surface will form droplets under the action of the interfacial tension at the gas–liquid–solid three-phase interface, and the droplets will form a contact angle θ at the gas–liquid–solid three-phase interface. When equilibrium is reached, the wetting formula can be obtained.

$$\sigma_{g-s}=\sigma_{l-s}+\sigma_{g-l}cos\theta .$$

The contact angle θ can judge the wetting of the surface. The smaller the contact angle, the better the hydrophilic performance. The *m*-HPAE solution was prepared with a concentration of 400 mg/L to fully soak the rock sample and then dried to obtain the processed rock sample, and a contact angle tester was used to measure the contact angle of the rock sample surface before and after the treatment.

In the process of water flooding, the migration of oil and water in the rock pores will be affected by the wettability of the rock. The wettability of the pores directly affects the efficiency of water flooding. The capillary resistance formed by the lipophilic pores will reduce the recovery. The capillary force formed by hydrophilic pores can be used as a driving force to improve oil recovery. When the oil-in-water emulsion system flows through the bottom layer, the more hydrophilic the surface of the rock, the lower will be the flow resistance. If the surfactant can change the originally hydrophobic rock surface into a hydrophilic rock surface, it will convert the capillary force from resistance to motive force and increase the oil recovery.

### Rheology

There are polymer chains in the *m*-HPAE structure, which will affect the extension structure in a high-concentration salt solution, and the formation environment is often accompanied by high salinity. This experiment explores the influence of salinity on the rheology of *m*-HPAE. An *m*-HPAE solution was prepared with a concentration of 400 mg/L, and NaCl was added to make its concentration reach 1000 mg/L and 10000 mg/L. The viscosity at different shear rates was measured by using a Brookfield rotary viscometer.

### Core Flooding Experiment

Two binary surfactant systems, namely, *m*-HPAE/sodium dodecylsulfate (LAS) and *m*-HPAE/NaOH, were prepared, and the interfacial tension was measured prior to the core flooding experiments. The viscosity of the crude oil (obtained from Daqing Oilfield) is 10cP at 45°C, and the salinity of the formation water is 3 × 10^3^ mg/L. The interfacial tension was measured using a TX-500C interfacial tensiometer at 500 rpm, and the data were recorded every 30 mins until the difference between the two consecutive readings was less than 0.001.

**FIGURE 4:**
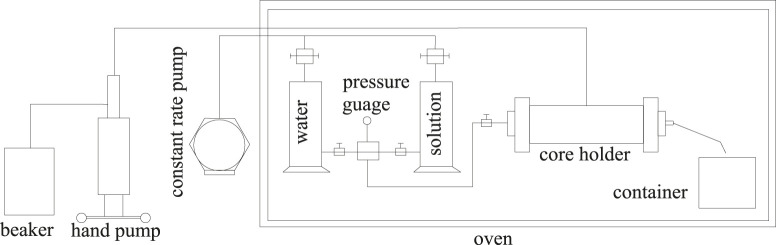
Core flooding experiment system.

The core flooding experimental setup is composed of pressure sensors, the core holder and the sample collecting container, and the hand pump and the constant rate pump (Figure 4).

All components were placed in an oven with the temperature set at 65°C, except the hand pump and the constant rate pump. The core was vacuumed and saturated with formation water at the atmospheric temperature and then with the crude oil at 65°C. The core was flooded by water at the same temperature until 98% water cut and the oil recovery factor by water flooding were calculated. Then, 0.3 pore volume of the surfactant solution was injected followed by water flooding until 98% water cut and the oil recovery factor by surfactant flooding were calculated. The injection rate was constant at 0.3 ml/min, and pressure data were recorded every 30 min.

## Results and Discussion

### Influential Factors on Syntheses

The processes where the intermediate and the final product are produced belong to the esterification and carboxymethylation reactions, respectively. The conversion rate in the esterification reaction is evaluated by the acid number which is the mass of potassium hydroxide (mg) required to neutralize the chemical (1 g). The conversion rate in the carboxymethylation reaction is calculated based on the hydroxyl value, which is potassium hydroxide (mg) required to neutralize the acetic acid taken up on acetylation of the chemical substance (1 g) that contains free hydroxyl groups.

As esterification reaction proceeds slowly at low temperature, and we recorded the data from the reaction conducted at 100°C. More intermediates are produced as the temperature increases. When the temperature was elevated to 115°C, the color of the product is light yellow, close to the color of the intermediate. The color of the product turns dark at a temperature above 130°C, indicating that the structure of the intermediate is broken, and an unexpected substance is produced.

**FIGURE 5:**
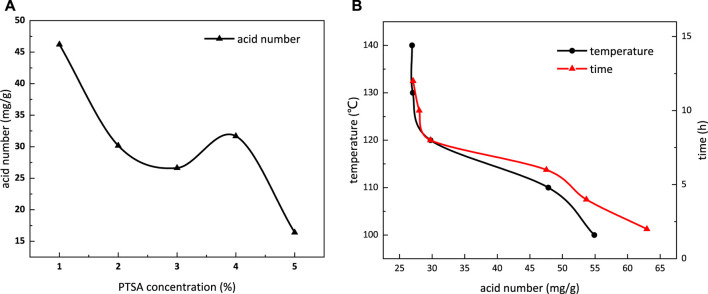
Influence of PTSA concentration **(A)** and temperature and reaction time **(B)** on the acid number in the esterification reaction.

There were no catalysts required in the carboxymethylation reaction, while various concentrations of PTSA (1, 2, 3, 4, and 5%) were used to accelerate the esterification reaction, since it is a reversible reaction. The reaction temperature was set at 120°C, reaction time was 8 h, and molar ratio of the intermediate and TMP was 9:1. The relationship between the acid number and concentration of PTSA is demonstrated in Figure 5A. The acid number decreases when PTSA concentration is increased till 3%. The acid number is determined to be 26.62 mg/g at 3% concentration, and in the meanwhile, the product exhibits light yellow color and viscous property. The reaction proceeds toward the AB_2_ intermediate, and the carboxyl groups are continuously consumed. The effects of PTSA itself on the acid number are neglected, considering its relatively small usage. The acid number of the system is abnormal when PTSA concentration is more than 4%. It indicates that some unexpected byproducts are produced by increasing the concentration of the catalyst. PTSA concentration is determined to be 3% in the esterification reaction to avoid byproducts during the syntheses of *m*-HPAE.

Figure 5B shows the influence of temperature and reaction time on the acid number in the esterification reaction. Acid number can be used to indirectly demonstrate the extent of the reaction. It decreases with the increasing temperature due to the fact that the hydroxyl groups are reacting with the carboxylic acid in the previously formed monomer. The stabilized acid number indicates reaction equilibrium at 120°C, which is also the temperature limit of the light-yellow intermediate being produced. The influence of various reaction times was also evaluated at 120°C, where 3% PTSA as the catalyst and 9:1 M ratio of the intermediate and TMP were used. The acid number presents a stabilized trend after the 8-h reaction, which indicates that the esterification reaction achieves equilibrium at 8 h. Therefore, in the esterification reaction, the temperature is determined to be 120°C, and the reaction time is 8 h.

**FIGURE 6:**
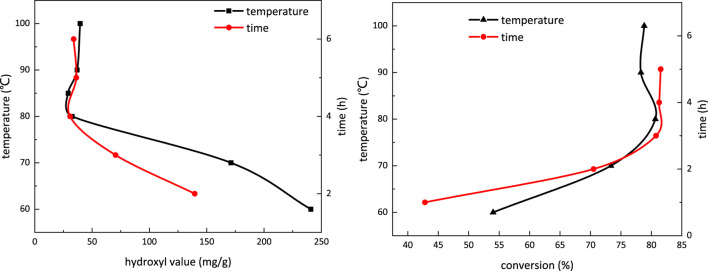
Influence of temperature and reaction time on the hydroxyl value **(A)** and conversion **(B)** in the carboxymethylation reaction.

Figure 6A depicts the influence of temperature and reaction time on the hydroxyl value in the carboxymethylation reaction. The hydroxyl value decreases with the increasing temperature and becomes stable at temperature above 80°C. With extended reaction time, the hydroxyl value also decreases and becomes stable when the reaction time is more than 4 h. Therefore, in the carboxymethylation reaction, the temperature is determined to be 80°C, and the reaction time is 4 h.

**FIGURE 7:**
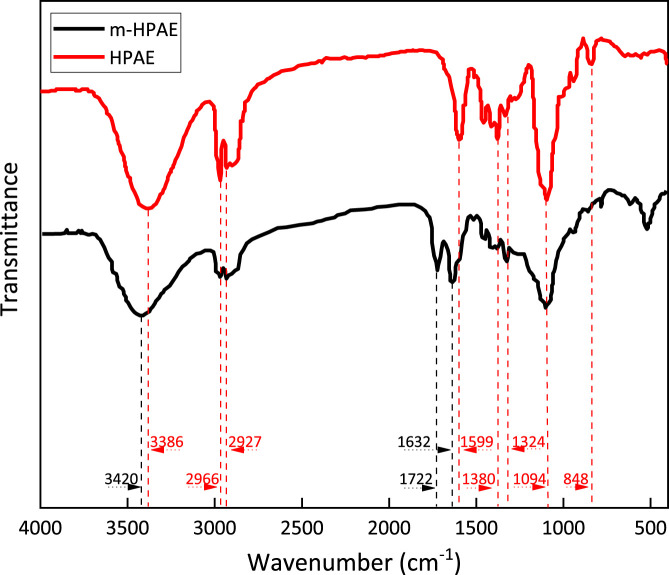
FT-IR of HPAE and modified HPAE (*m*-HPAE).

Figure 6B shows the influence of temperature and reaction time on conversion in the carboxymethylation reaction. When the temperature is above 80°C and reaction time is over 4 h, the conversion trend in the carboxymethylation reaction does not present obvious change, which is in line with the conclusion given in Figure 7. The conversion at 80°C reaction for 4 h is 80.77%.

### FT-IR

The spectral data of HPAE and the modified HPAE (*m*-HPAE) were recorded by using a FT-IR spectrometer in a wide spectral range, as is shown in Figure 7.

The strong absorbance bands at 3,420 cm^−1^ (*m*-HPAE)and 3,386 cm^−1^ (HPAE) indicate that the -OH group is present in the molecular structure. In the process of the hydroxylation reaction of HPAE, -COONa is formed, and the terminal–OH is consumed, resulting in a less-intense absorption band. Meanwhile, it is normal that the amide–hydroxyl bonding peak appears in this region. The saturated carbon chain is elongated as a result of the hydroxylation of HPAE. This largely weakens the electron-withdrawing effect of adjacent–OH on amide groups, resulting in the enhanced inductive effect of adjacent carbon carbonyl C=O oxygen atoms on amide groups. That explains why the absorption peak has shifted to a higher wavenumber (3,420 cm^−1^).

The absorption bands at 2,966 cm^−1^ and 2,927 cm^−1^ correspond to CH^3-^ and CH_2_- in the molecule, respectively. The C–H absorption band at 2,890 cm^−1^ with low intensity is associated with the saturated carbon chain structure in the molecule.

Both HPAE and *m*-HPAE molecules show strong -COO- antisymmetric stretching vibration at 1,599 cm^−1^. This absorption band is not associated to the amino acid (-CONH-) -COO- structure because the intensity is not highest. The presence of the -COO- structure in HPAE molecules throughout the modification processes is a strong evidence that the branched structure is built from the esterification reaction of HPAE and *m*-HPAE.

There are multiple strong absorption bands in the *m*-HPAE spectrum. 1722 cm^−1^ and 1,632 cm^−1^ correspond to ester carbonyl and tertiary amide carbonyl groups, respectively, which proves that the molecular structure at the position of the end group is modified to be –CO–N–COO-. Meanwhile, the carboxylic acid radical is present as indicated by the weak but characteristic absorption band at 550 cm^−1^ (-COO- vibration).

Multiple weak absorption bands are detected in the range of 1,500–1,000 cm^−1^ for both HPAE and *m*-HPAE, respectively. 1,380 cm^−1^ and 1,324 cm^−1^ correspond to amide C–N, while the broad absorption band at 1,094 cm^−1^ is connected to the aliphatic amide and C–O–C structure. The broad shape of the absorption band results from the increased contents of C–O–C by *m*-HPAE modification.

In theory, the products synthesized in this experiment can only be hyperbranched products or monomeric self-polymerized products. HPAE and *m*-HPAE are successfully obtained, and the expectation of the product design is met, as verified by infrared spectroscopy. The presence of hyperbranched and carboxymethylated products indicates the success of the branching and the carboxymethylation reactions. This is also confirmed by the increase in the value of the hydroxyl group.

### Surface Tension

**FIGURE 8:**
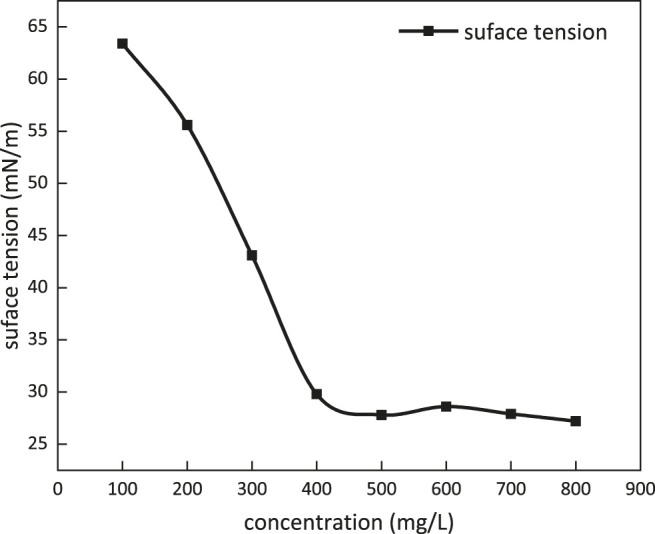
Surface tension dependence on the concentration of *m*-HPAE.

Surface tension dependence on the concentration of *m*-HPAE at 25°C is given in Figure 8 *m*-HPAE is able to lower surface tension to around 28 mN/m, exhibiting excellence compared to the conventional surfactants such as OP-10 (around 31 mN/m) and LAS (around 32 mN/m).

### Critical Micelle Concentration and Krafft Point

**FIGURE 9:**
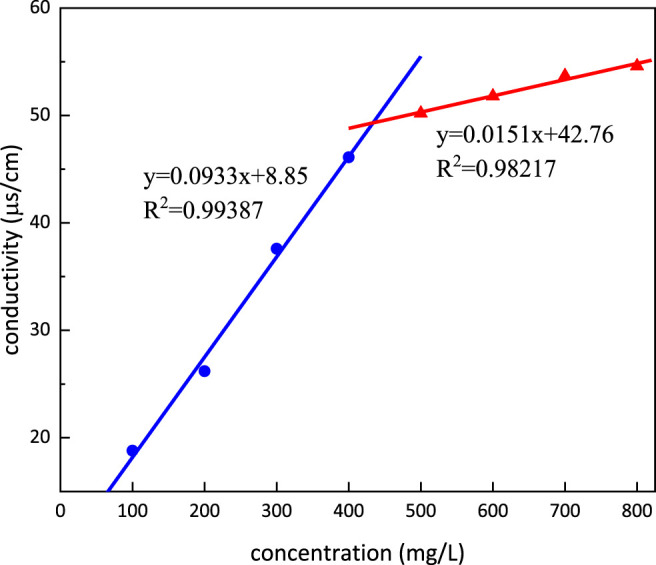
Conductivity dependence on the concentration of *m*-HPAE.

Conductivity dependence on the concentration of *m*-HPAE is shown in Figure 9. The conductivity was increasing with the increasing concentration of *m*-HPAE; however, the rate of the increase was different presenting a turning point (intersection of both lines) in the trend. This turning point, namely, CMC, is calculated to be 433.63 mg/L. The Krafft point is determined to be 5°C, which means that it can be used under room temperature conditions.

### Adsorption Property

**FIGURE 10:**
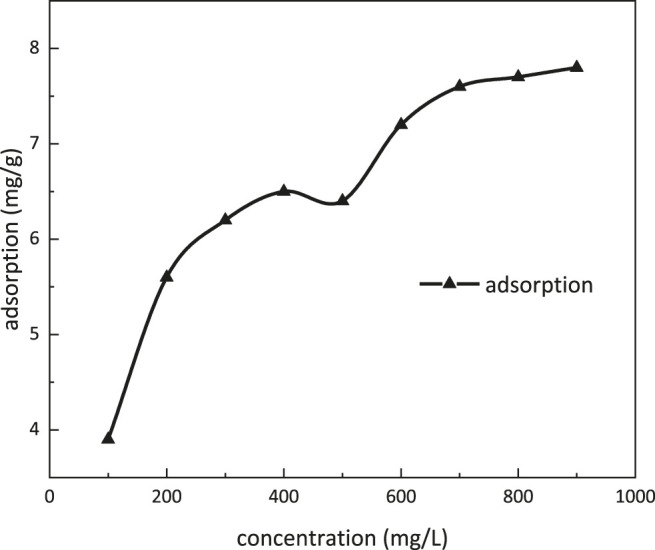
Adsorption dependence on the concentration of *m*-HPAE.

Adsorption dependence on the concentration of *m*-HPAE is given in Figure 10. The adsorption was increasing with increasing concentration of *m*-HPAE. In the range of 400–500 mg/L concentration, the adsorption onto the oil sand surface achieved a stable value. The CMC was also within this range, indicating that the adsorption equilibrium was achieved at the CMC. When the concentration was above 500 mg/L, the adsorption started to increase, breaking the stabilization. It is well-known that the dual layers of electrodiffusion were formed at the surface of the oil sands when contacting an aqueous solution. The negative ions in the surfactant solution were opposite to the electric charge on the surface of the oil sands, resulting in reduction in electrostatic action and weakening in the dual layers of electrodiffusion. More surfactant monomers tend to seek adsorption vacancy at higher concentration.

### Micellar Solubilization

**FIGURE 11:**
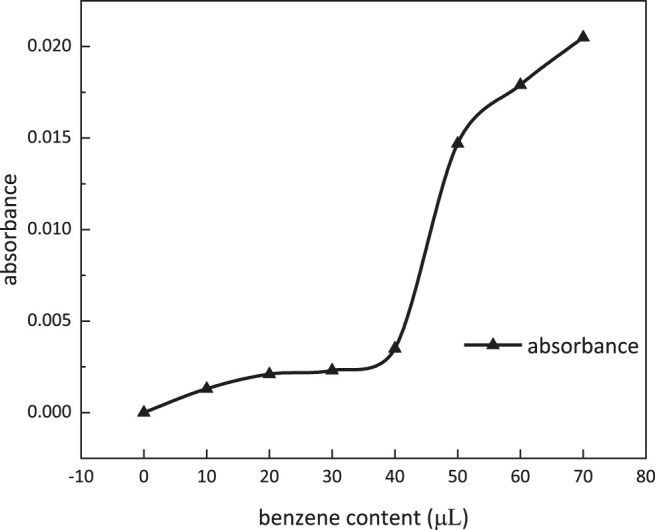
Absorbance dependence on benzene content.

Absorbance dependence on benzene content at a wave number of 560 nm is shown in Figure 11. When benzene content was more than 40 μl, the absorbance of the solution was increasing dramatically. Micellar solubilization was determined to be 600 ml/mol at 40 μl of benzene content.

### Wettability

**FIGURE 12:**
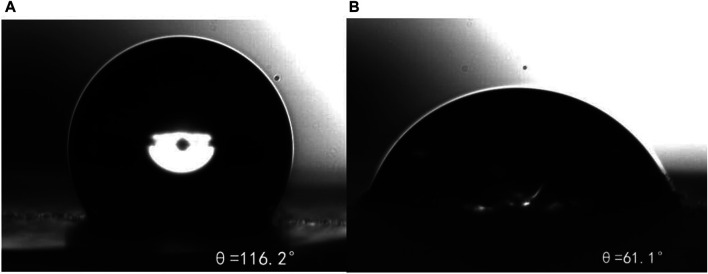
Contact angle of hydrophobic formation of the rock sample **(A)** and rock sample after *m*-HPAE treatment **(B)**.

Figure 12 shows that the contact angle (a) of the untreated rock sample is 116.2°, and the contact angle (b) after treatment is reduced to 61.1°, indicating that *m*-HPAE adsorbed on the rock surface can effectively improve the hydrophilicity of the rock surface, reduce the flow resistance of oil in water fluid in the water flooding system, reduce the possibility of blockage during the migration of formation of lipophilic particles, and achieve the purpose of improving formation permeability and enhancing oil recovery.

### Rheology

**FIGURE 13:**
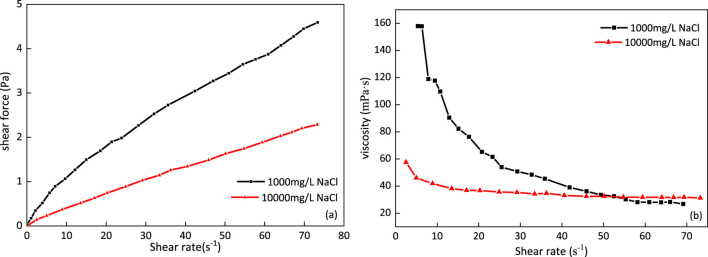
Rheology of the *m*-HPAE solution in 1,000 mg/L and 10,000 mg/L salt solution.

Figure 13 shows that the apparent viscosity of the *m*-HPAE solution decreases with the increase in salinity. After the -COONa group is ionized in the solution, the -COO^-^ group is found to be electronegative. The mutual exclusion between groups results in the molecular chain being more stretched, the flow resistance increases, and the viscosity increases. When the high-concentration salt solution is added to the system, the cations gather around the electronegative groups, shielding the effective charge, reducing the anion mutual exclusion, shrinking the molecular chain structure, and reducing the flow resistance and viscosity. The m-HPAE solution maintains the shear thinning characteristic when adding the low-concentration salt solution (1000 mg/L), while after adding the high concentration salt solution (10000 mg/L), the shear thinning effect decreases with the increase of the shear rate, which is similar to Newtonian fluid, and the viscosity is maintained near 31 MPa s.

### Core Flooding

**FIGURE 14:**
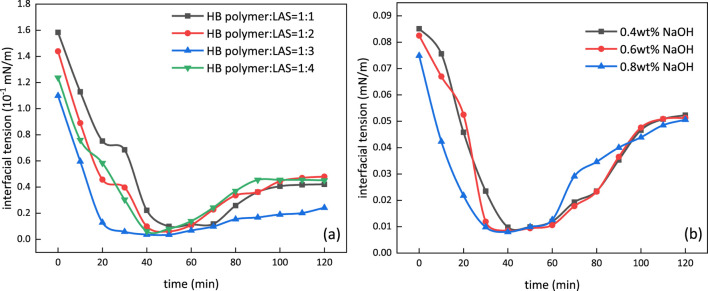
Interfacial tension in various formulations of *m*-HPAE/LAS **(A)** and *m*-HPAE/NaOH **(B)**.

The formulation of *m*-HPAE/LAS and *m*-HPAE/NaOH with various ratios was prepared, and the interfacial tension was measured using a TX-500C interfacial tensiometer, as shown in Figure 14. Figure 14A demonstrates the interfacial tension trend with various mass ratios of *m*-HPAE/LAS. The interfacial tension can be lowered to a level of 10^–2^ by individual *m*-HPAE or LAS; however, this value was further lowered to a level of 10^–3^ using the formulation of *m*-HPAE/LAS due to the synergistic effect exerted by petroleum sulfonate and the HB carboxylate surfactant. The negatively charged oxygen atoms in sulfonic groups enhanced the interaction with crude oil by making full use of anionic surfactants’ advantage of maximum interfacial adsorption, leading to a lower interfacial tension at a relatively low usage. Being formulated with *m*-HPAE, this advantage was further exhibited to achieve the optimum effect with least usage. The interfacial tension was lowered to 3.5 × 10^–3^ mN/m at 1:3 mass ratio of *m*-HPAE/LAS.

Figure 14B depicts the interfacial tension change with different mass fraction of NaOH in 0.04 wt% *m*-HPAE. The interfacial tension was also lowered to a level of 10^–3^ using the formulation of *m*-HPAE/NaOH, and the three mass fractions of NaOH did not present obvious difference. The reduction in interfacial tension was caused by competitive adsorption of *m*-HPAE and the alkali. The active materials were also produced by the reaction between the alkali and the acid in the crude oil, resulting in further reduction in interfacial tension.

Due to the well-known reservoir damage caused by alkali, it is not recommended to be used for flooding. The LAS was proven to be able to lower interfacial tension to the same level as NaOH when being formulated with *m*-HPAE; therefore, only the binary surfactant system of *m*-HPAE/LAS was used in core flooding experiments. The *m*-HPAE flooding and water flooding experiments were also conducted for comparison. The permeability of heterogeneous cores ranged from 100 mD to 1000 mD. 0.04 wt% *m*-HPAE and 1:3 mass ratio of *m*-HPAE/LAS were used in the flooding experiments. The results of *m*-HPAE flooding and *m*-HPAE/LAS flooding are listed in Table 1 and Table 2, respectively.

**TABLE 1:** *m*-HPAE flooding results.

Core No	K (mD)	S_w_ (%)	E_w_ (%)	E_1_ (%)	E (%)
1	129.85	60.87	31.98	10.40	42.38
2	269.65	42.55	29.84	13.87	43.71
3	604.91	58.46	31.94	21.58	53.52
4	701.55	52.69	38.05	20.64	58.69
5	819.68	68.87	34.99	18.98	53.97
6	921.66	62.35	32.69	22.58	55.27
7	1,021.87	70.15	41.58	25.15	66.73

**TABLE 2:** *m*-HPAE/LAS flooding results.

Core No	K(mD)	S_w_ (%)	E_w_ (%)	E_1_ (%)	E (%)
8	127.23	57.26	18.65	27.69	46.34
9	268.97	60.21	22.64	22.34	44.98
10	603.42	74.98	28.23	21.35	49.58
11	713.52	69.31	39.43	19.36	58.79
12	823.65	72.12	34.86	29.64	64.50
13	918.85	63.86	27.86	32.21	60.07
14	1,054.36	70.23	43.32	27.56	70.88

As shown in Table 1, the oil recovery was further enhanced by *m*-HPAE flooding after 98% water cut was observed in water flooding. The increment was ranging from 10.4 to 25.15% in the group experiments. Two aspects of the HPB contribute to enhanced oil recovery. On the one hand, *m*-HPAE, as described above being a surfactant, is able to lower interfacial tension to a great extent and on the other hand, the *m*-HPAE structure with an inward lipophilic group and many outward hydrophilic groups creates the perfect space for the migration of the oil drops.

As is given in Table 2, oil recovery was further enhanced by *m*-HPAE/LAS following water flooding, ranging from 19.36 to 32.21%. The formulation made full use of the combined chemicals, namely, polymer, for water inflow profile modification to enhance sweep efficiency and surfactants for lowering interfacial tension. The synergistic effect resulted in the formulation being more efficient, especially in low permeability cores, than the single use of *m*-HPAE.

## Conclusion

*M*-HPAE was synthesized through two-step reactions, namely, esterification and carboxymethylation, to form the intermediate and the final product, respectively. The optimum condition for esterification was 8 h at 120°C, where 3% PTSA as the catalyst and 9:1 mol ratio of the AB_2_ monomer and TMP were used. The optimum condition for carboxymethylation was 4 h at 80°C, and the ratio of the carboxymethylation reagent to HPAE was 1:12.

The properties of *m*-HPAE were evaluated experimentally and determined to be more excellent than the conventional surfactants. The CMC was 433.63 mg/L, the Krafft point was 5°C, and the surface tension was lowered to 28 mN/m. In the range of 400–500 mg/L concentration, the adsorption onto the oil sand surface achieved equilibrium and micellar solubilization reached 600 ml/mol.

The contact angle of core samples treated with *m*-HPAE decreased from 116.2° to 61.1°, which can change from the hydrophobic surface to the hydrophilic surface, indicating that *m*-HPAE can promote the migration of oil in water emulsion.

Rheological experiments show that the *m*-HPAE solution maintains the characteristics of shear thinning in the environment of the low-concentration salt solution, while its apparent viscosity further decreases under the action of the high-concentration salt solution. With the increase in the shear rate, the viscosity tends to a fixed value and the property is close to Newtonian fluid.

The interfacial tension can be lowered to a level of 10^−2^ mN/m by the single use of *m*-HPAE, and the value further decreased to a level of 10^−3^ mN/m while being formulated with LAS or NaOH. The oil recovery of water flooding was further enhanced by the single use of *m*-HPAE or the combination of *m*-HPAE/LAS. The latter exhibited more prosperous advantages in low-permeability reservoirs.

## Data Availability

The original contributions presented in the study are included in the article/Supplementary Material; further inquiries can be directed to the corresponding author.

## Nomenclature

HBP: Hyperbranched polymer
HPAE: Hyperbranched polyurethane
sCMC: Critical micelle concentration
K: Permeability
P: Porosity
S_w_: Water saturation
E_w_: Oil recovery by water flooding
E_1_: Oil recovery by *m*-HPAE flooding
E_2_: Oil recovery by *m*-HPAE/LAS
E: Overall oil recovery
